# A-to-I RNA edited POLA2 attains carcinogenesis in prostatic cancer by impeding immune infiltration and upregulating BTBD7

**DOI:** 10.1007/s12672-025-02449-8

**Published:** 2025-05-14

**Authors:** Shengyi Lin, Hang Huang, Yeping Li, Yongyong Lu, Tingyu Ye

**Affiliations:** Department of Urology, The First Affiliated Hospital of Wenzhou Medical University, Wenzhou, 325000 China

**Keywords:** A-to-I RNA editing, POLA2, Prostatic cancer, BTBD7, Immune infiltration, Glycolysis

## Abstract

**Background:**

Recently, prostate cancer (PCa) has been increasing in incidence and mortality, which seriously threatens men’s physical and mental health. Adenosine (A)-to-inosine (I) RNA editing, contributing to nearly 90% of all editing events in human, has been reported to contribute to pathogenesis and progression of cancer. Here, we aimed to elaborate the role and mechanism of A-to-I-edited POLA2 in PCa.

**Methods:**

RT-qPCR, Western blotting, and immunohistochemistry were used to assess gene expression. RNA editing levels were determined by Sanger sequencing. Colony formation, CCK-8, and Transwell assays were conducted to detect cell proliferation and metastasis. And Flow cytometry assay was applied to examine CD8+ T cell activity and tumor cell apoptosis. Dual-luciferase reporter assay demonstrated the relationship between gene and miRNA. The ability of glycolysis was measured by Seahorse XF96 Analyzer.

**Results:**

A-to-I RNA overediting of POLA2 was identified in PCa patients, which was related to unfavorable clinical outcomes and prognosis. The A-to-I RNA editing of POLA2 was mediated by ADAR1 enzyme in human cancers. Functionally, A-to-I RNA editing endowed POLA2 with carcinogenicity in PCa development, and POLA2 overediting aggravated cell viability and metastasis of PCa. More importantly, POLA2 overediting fortified glycolysis and impaired CD8+ T cell cytotoxicity in PCa. Mechanically, edited POLA2 upregulates BTBD7 expression in PCa by binding to miR-596.

**Conclusion:**

A-to-I RNA edited POLA2 attained carcinogenesis in PCa by impeding immune infiltration, fortifying glycolysis and upregulating BTBD7, indicating that edited POLA2 has the potential to become a tool for gene therapy.

## Introduction

The most common malignancy of the male genitourinary system is prostate cancer (PCa), the incidence of which increases with age [1]. In recent years, the incidence of PCa has been rising all over the world. According to statistics, approximately 1.3 million new cases and 359,000 deaths were reported worldwide in 2018, accounting for 13.5% of all male malignant tumors [2]. The mortality rate of PCa accounts for 6.7% of the mortality rate of male malignant tumors, ranking fifth [3]. The incidence and mortality of PCa have obvious regional differences. While PCa is more common in developed countries like the United States, it is less prevalent in Asian nations [4]. PCa is becoming more prevalent in China each year. And currently PCa ranks the third in male malignant tumors in China, which is one of the important reasons for the death of male cancer patients [5]. In addition, PCa is a kind of cancer with particularly slow progress, and it is not easy to find in the early stage. However, once PCa begins to grow rapidly or spread outside the prostate, it will threaten the survival of patients [6]. Therefore, it is of great value to explore the important influencing factors affecting PCa progression for further understanding the pathogenesis of PCa.

RNA editing is a process of inserting, deleting or replacing single or multiple nucleotides in the initial transcript, thus changing the original genetic information [7]. Currently, there are many studies focusing on RNA editing about two types of base substitution, namely, cytosine (C)-to-uracil (U) RNA editing events and Adenosine (A)-to-inosine (I) RNA editing events [8–11]. Recently, there has been considerable interest in the editing phenomenon of A-to-I RNA in mammals. It refers to the process of ADAR editing enzyme specifically recognizing double-stranded initial transcripts, and deamination of adenine (A) at some specific sites to form hypoxanthine (I). Then, I is recognized as guanine (G) in the process of base pairing, which causes the change of genetic information. At present, the role of A-to-I RNA editing events in various human diseases is increasingly being recognized. For example, A-to-I-edited miR-1251-5p restricted lung adenocarcinoma tumor growth and metastasis [12]. In developing sevoflurane neurotoxicity, ADAR1 prevented PANoptosis due to A-to-I RNA editing [13]. By triggering AZIN1 A-to-I editing, inflammation primed the murine kidney for recovery [14]. In androgen-sensitive PCa cells, miR-379 editing minimizes the growth-suppressing effects of unedited miR-379, promoting tumor growth [15]. However, the study about A-to-I RNA editing events including edited POLA2 in PCa is rare.

POLA2 has been implicated in a wide variety of tumor types (breast cancer, bladder cancer, glioblastoma multiforme, gastrointestinal stromal tumor, non-small cell lung cancer, ovarian cancer) and serves as a prognostic marker for patients. Specifically, Dang et al. showed that glioblastoma multiforme manifested significantly increased expression of POLA2, which was associated with poorer survival rate [16]. Cao et al. also found that POLA2 can be used as a carcinogen of bladder cancer to promote tumor angiogenesis and cell division [17]. More importantly, POLA2 has been found to regulate immune infiltration in human cancers [18]. Immune infiltration of tumor cells is a defensive response, which means that the body removes tumor cells by recruiting various immune cells such as T cells and macrophages [19]. Moreover, A-to-I RNA editing events have been demonstrated to regulate tumor immune infiltration. In hepatocellular carcinoma, co-editing of A-to-I RNA predicted clinical outcome and was related to the infiltration of immune cells [20]. However, it is remains unknown whether A-to-I RNA edited POLA2 is involved in PCa development by affecting immune infiltration.

Mechanically, this study found that BTBD7 is a downstream target of edited POLA2 in PCa. It has been reported that BTBD7 inhibits the adhesion between cells by repressing “cadherin”, thus releasing cells [21]. Therefore, many studies have shown that BTBD7 can trigger epithelial-mesenchymal transition (EMT) in human cancer by modulating the expression of E-cadherin and N-cadherin [22]. More importantly, Chen et al. has been proved that BTBD7 accelerated the EMT, invasion and proliferation of PCa cells [23]. But the regulatory mechanism of edited POLA2/BTBD7 has not been reported in previous studies. Therefore, our research not only analyzed the function of edited POLA2 and its influence on immune infiltration, but also preliminarily analyzed the regulatory mechanism of edited POLA2/BTBD7 in PCa. Our study will further enrich the theoretical research on the pathogenesis of PCa and provide a new direction for its diagnosis and treatment.

## Materials and methods

### Patients and specimens

There are 38 PCa patients from Shandong (SD) and 37 PCa patients from Zhenjiang (ZJ) involved in this study. This study was conducted in accordance with Helsinki Declaration and approved by the ethics committee of The First Affiliated Hospital of Wenzhou Medical University. Surgically resected tumor tissues from PCa patients were stored at − 80 °C in a refrigerator (TDE30086FV-ULTS, Thermo Fisher Scientific Inc, Waltham, MA, USA). Transurethral prostate resection patients with benign prostatic hyperplasia were recruited for the collection of normal tissues. Participant consent was obtained before the study began.

### Cell culture and transfection

Human PCa cell lines DU145 and LNCap (Chinese Academy of Sciences, Shanghai, China) were incubated with RPMI-1640 medium (Gbico, USA) containing 10% FBS, streptomycin and penicillin (Invitrogen, Carlsbad, CA) in an incubator with 5% CO_2_ at 37 °C.

Wild type (wt) or edited (ed) POLA2 vector and miR-596 mimics or inhibitor (Genechem, Shanghai, China) was transfected into PCa cells using LipofectamineTM3000 (Invitrogen, Carlsbad, CA). A control group (NC) was set up with PCa cells without transfection.

### ADAR perturbation experiment

First, the mutations of ADAR1 and ADAR2 were generated by Site-directed mutagenesis. After mutation, we found that mutant ADAR1-E912A and ADAR2-E396A lost the editase activity. Then, DU145 and LNCap cells were transfected with GFP, ADAR1-E912A, WT-ADAR1-WT, ADAR2-E396A or WT-ADAR2 expression vectors, respectively. After incubation, POLA2 editing level in above transfected PCa cells was evaluated by Sanger sequencing (RiBobio, Guangzhou, China).

### RT-qPCR

The extraction of total RNA in clinical specimens and PCa cells was performed with TRIzol reagent (Invitrogen). PrimeScript RT Master Mix and SYBR Premix ExTaq (Takara, Otsu, Japan) were employed to conduct synthesis of cDNA and qPCR with 7500 real-time PCR System. The 2^−ΔΔCT^ method was used to quantify relative gene expression, with GAPDH serving as the endogenous control.

### Colony formation assay

Transfected PCa cells were plated in 6-well plates and cultured at 37 °C for 2 weeks. The colonies were stained with 0.1% crystal violet after fixation with paraformaldehyde. The number of colonies was capture and counted.

### EdU (5-Ethynyl-2ʹ-deoxyuridine) assay

The Cell Light EdU DNA imaging kit (RiboBio, Guangzhou, China) was used for the EdU assay. Transfected PCa cells plated in 96-well culture plates were incubated for 12 h. Next, the cells treated with a 10 μM EdU solution were incubated for 2 h at 37 °C before being fixed. The nuclei of the fixed cells were stained with DAPI for 30 min following fixation with 4% paraformaldehyde. Finally, fluorescence microscope (Carl Zeiss, Germany) was used to visualize and quantify EdU-positive cells.

### Transwell assay

The 8 μm diameter Transwell chamber (Corning Incorporated, Corning, NY, USA) with or without Matrigel (BD Biosciences) was used for cell invasion and migration. DU145 and LNCap cell suspension (200 μl) was put in the upper chamber, while RPMI-1640 medium (FBS) was added in the lower chamber. After cultured for 24 h, the moving cells were fixed with 4% paraformaldehyde and stained with 0.1% crystal violet. Image capture and counting of moving cells was conducted under the microscope at an original magnification of 200×.

### Animal experiments

The animal experiment was approved by the Animal Care Committee of The First Affiliated Hospital of Wenzhou Medical University. BALB/c nude mice (5 weeks old) were purchased from SLAC Laboratory Animal Co., Ltd. (Shanghai, China). Injections of DU145 cells with wt-POLA2 or ed-POLA2 (2 × 10^6^) were made subcutaneously in the hindlimbs of BALB/c nude mice. Mice without injection were set as Blank group. In an SPF animal house, the mice were housed at 26–28 °C and 40%–60% humidity. Tumor volume (mm3) was examined every week. We euthanized the mice after 5 weeks to obtain tumor tissue for further studies.

### Immunohistochemistry assay

Mice tissues were paraffin-embedded and sliced into 4-mm thick sections. BTBD7 E-cadherin, N-cadherin and Ki67 primary antibodies (Abcam, USA) were used to incubate the sections overnight at 37 °C for 2 h after dewaxing, antigen repair and blocking. After that, secondary antibodies were incubated at room temperature with the sections. After that, the slices were incubated with HRP-labeled streptavidin for 10 min at 37 °C. Hematoxylin staining solution was added until the tissue is completely covered. After sealing and drying, sections were observed under a microscope.

### Lactate production, glucose consumption and glucose metabolism analysis

Lactate Assay Kit and Glucose Assay Kit (Abcam, USA) were used for the detection of lactate and glucose levels in DU145 cells. The ability of glucose metabolism was assessed via the oxygen consumption rate (OCR) and extracellular acidification rate (ECAR) on Seahorse XF96 Analyzer (Seahorse Bioscience). Briefly, DU145 cells containing wt-POLA2 or ed-POLA2 vector in XF96 cell plate were incubated for 12 h. XF assay medium with 4.5 g/l glucose, 2 mM glutamine and 1 mM sodium pyruvate was used for OCR. Then, the of 1 μM oligomycin, 1 μM FCCP, and 1 μM rotenone were sequentially added for OCR detection. Additionally, 4.5 g/l glucose, 1 μM oligomycin and 50 mM 2-DG were sequentially added for ECAR detection in XF assay medium containing 2 mM glutamine.

### Isolation of CD8+ T cells

We isolated peripheral blood mononuclear cells (PBMCs) using Ficoll gradients from blood samples of healthy donors. MACS CD8+ T Cell Isolation Kit (Miltenyi Biotec, Germany) was used for CD8+ T cell purification from fresh PBMCs. After washing, CD8+ T cells were incubated in RPMI-1640 medium (10% FBS, 100 IU/ml IL2) and activated by the anti-CD3/CD28 monoclonal antibodies (mAbs).

### Flow cytometry assay

Tumor cell apoptosis was examined by using annexin V-fluorescein isothiocyanate/PerCP (Abcam, Shanghai, China) based on the manufacturer’s protocols.

The cellular grouping of immune cells in mice tumors was also performed by Flow cytometry assay. Briefly, CD16/32 antibody was firstly incubated with the isolated cells from mice tumors on ice for 15 min. The cells were then stained with CD45-APC/Cyanine7 (Biolegend, Clone30-F11, cat#103116), CD8-PE (Biolegend, Clone YTS156.7.7, cat#126608) and CD4-APC (Biolegend, Clone CloneGK1.5, cat#100434) antibodies for 30 min. Next, FACS buffer containing DAPI (1:2000) was added to resuspend the isolated cells for 10 min. Intracellular T cell cytotoxic molecules in CD8+ T cells were detected using Cell Stimulation Cocktail (eBioscience, cat#00-4970-93) and Brefeldin A (eBioscience, cat#88-8823-88). IFN-γ-AF700 (Biolegend, Clone XMG1.2, cat#505824) and Granzyme B-Percp/Cyanine5.5 (Biolegend, Clone QA16A02, cat#372212) antibodies were applied to stain CD8+ T cells for 30 min. Immune cells were acquired with BD Biosciences’ LSR Fortessa flow cytometer and analyzed with TreeStar’s Flowjo software.

### Bioinformatics analysis

The novel target miRNA of ed-POLA2 was predicted by CAeditome database (https://ccsm.uth.edu/CAeditome/index.html). The target gene prediction of miR-596 was analyzed by TargetScanHuman 7.2 (https://www.targetscan.org/vert_72/). The relations between POLA2 expression and abundance of tumor-infiltrating lymphocytes (TILs) were analyzed through TISIDB database (http://cis.hku.hk/TISIDB/).

### Dual-luciferase reporter assay

The 3ʹ-UTR of ed-POLA2, wt-BTBD7 or mut-BTBD7 containing the binding site of miR-596 was inserted into pmiRRB-Report™ vector (RiboBio), respectively. Next, miR-596 mimics and ed-POLA2 luciferase reporter vector were co-transfected into HEK293 cells. Additionally, miR-596 mimics and ed-POLA2 luciferase reporter vector with wt-BTBD7 or mut-BTBD7 were also co-transfected into HEK293 cells. After 24 h of incubation, Promega’s Dual-Luciferase Reporter Assay System Kit was used for luciferase activity detection.

### Western blot assay

The concentration of protein extracted by RIPA lysate (Beyotime) was examined using the PIERCE BCA protein assay kit (Thermo Fisher Scientific). After electrophoresis by 10% SDS-PAGE, the separated protein was transferred to PVDF membrane (Millipore, Billerica, MA, USA) and blocked with 5% skimmed milk. Next, the protein was incubated with ADAR1p150, ADAR1p110, ADAR2, E-cadherin, N-cadherin, vimentin and GAPDH primary antibodies (Abcam, USA) overnight at 4 °C, respectively. After removing primary antibodies, the corresponding secondary antibodies were added to incubate the protein for another 1 h at room temperature. Blots were developed using Enhanced Chemiluminescence Detection Kit (Thermo Fisher Scientific, Inc.).

### Statistical analysis

Statistical analysis was performed with GraphPad Prism 10.0 (San Diego, CA). Measured data are shown as mean ± SD. The difference between groups were compared using one-way ANOVA and T test. Pearson correlation method and Kaplan–Meier method were used for correlation and survival analysis. Statistical significance difference means P < 0.05.

## Results

### A-to-I RNA editing of POLA2 is related to unfavorable clinical outcomes and prognosis in PCa patients.

**Fig. 1:**
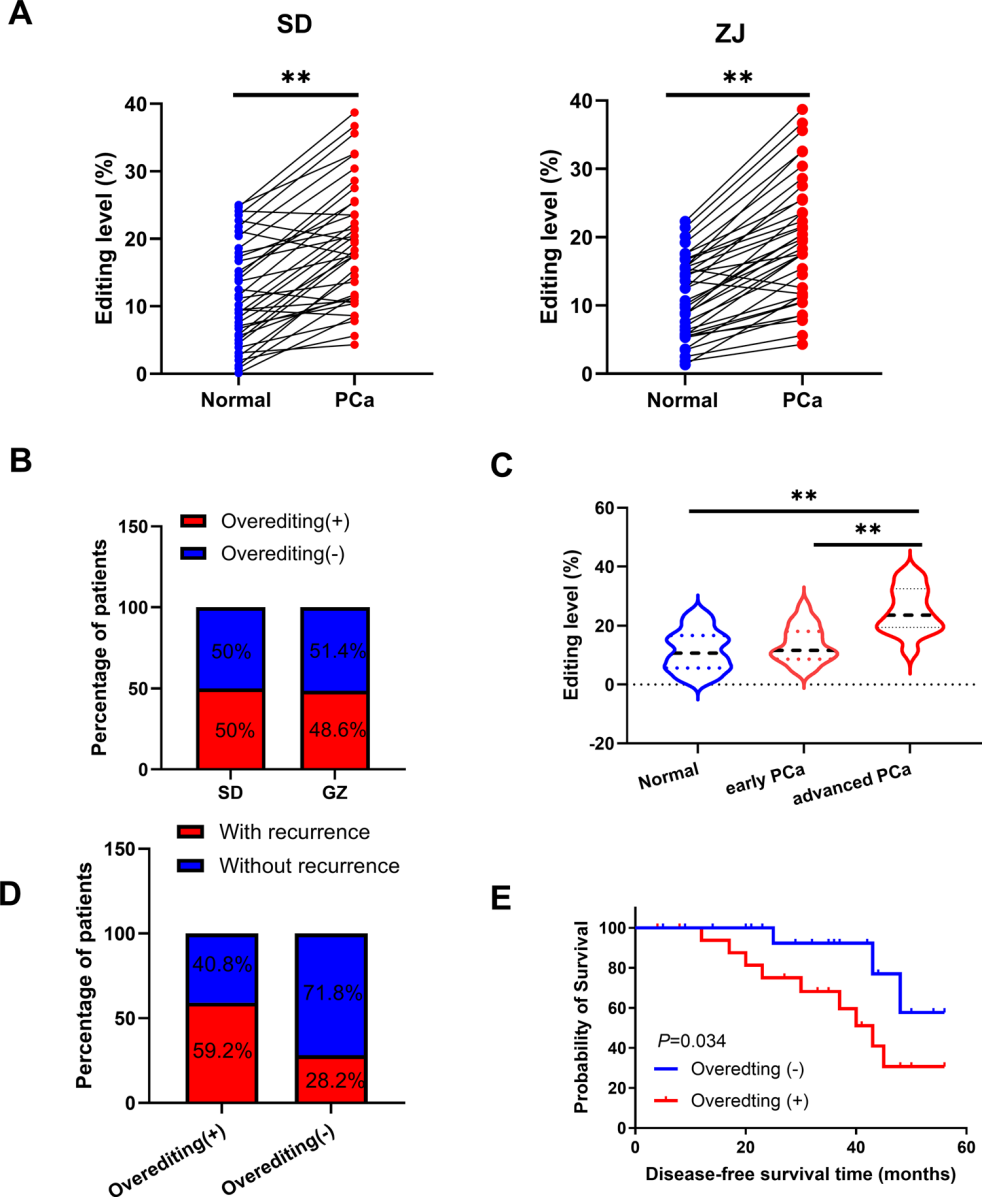
A-to-I RNA editing of POLA2 is related to unfavorable clinical outcomes and prognosis in PCa patients. **A** POLA2 editing in PCa and normal specimens from different cities. **B** The percentage of PCa patients with POLA2 overediting. **C** POLA2 editing in normal tissues and PCa tissues at different stage. **D** Association between recurrence incidence and POLA2 overediting. **E** Kaplan–Meier plots for the disease-free survival rate of PCa patients in the groups with (+) and without (−) overediting. **P < 0.01

First, we found that A-to-I RNA editing of POLA2 occurred in PCa based on CAeditome database (https://ccsm.uth.edu/CAeditome/index.html). After sequencing analysis, POLA2 overediting was identified in PCa tissues compared to normal tissues, and an overediting in tumors was defined by an increase of at least 10% in editing over normal tissue (Fig. 1A). Moreover, 50.0% (19/38 patients) and 48.6% (18/37 patients) of PCa patients from two different cities were identified to have POLA2 overediting event (Fig. 1B). Compared to PCa patients at early stage, higher editing level of POLA2 was found in PCa patients at advanced stages (Fig. 1C). Meanwhile, PCa patients with tumor recurrence had a higher frequency of POLA2 editing than those without (Fig. 1D). Additionally, there was a lower survival rate for PCa patients with POLA2 overediting than patients with low POLA2 editing level (Fig. 1E). The above results indicate that A-to-I RNA editing of POLA2 is related to unfavorable clinical outcomes and prognosis in PCa patients.

### A-to-I RNA editing of POLA2 is mediated by ADAR1 enzyme in cancers.

**Fig. 2:**
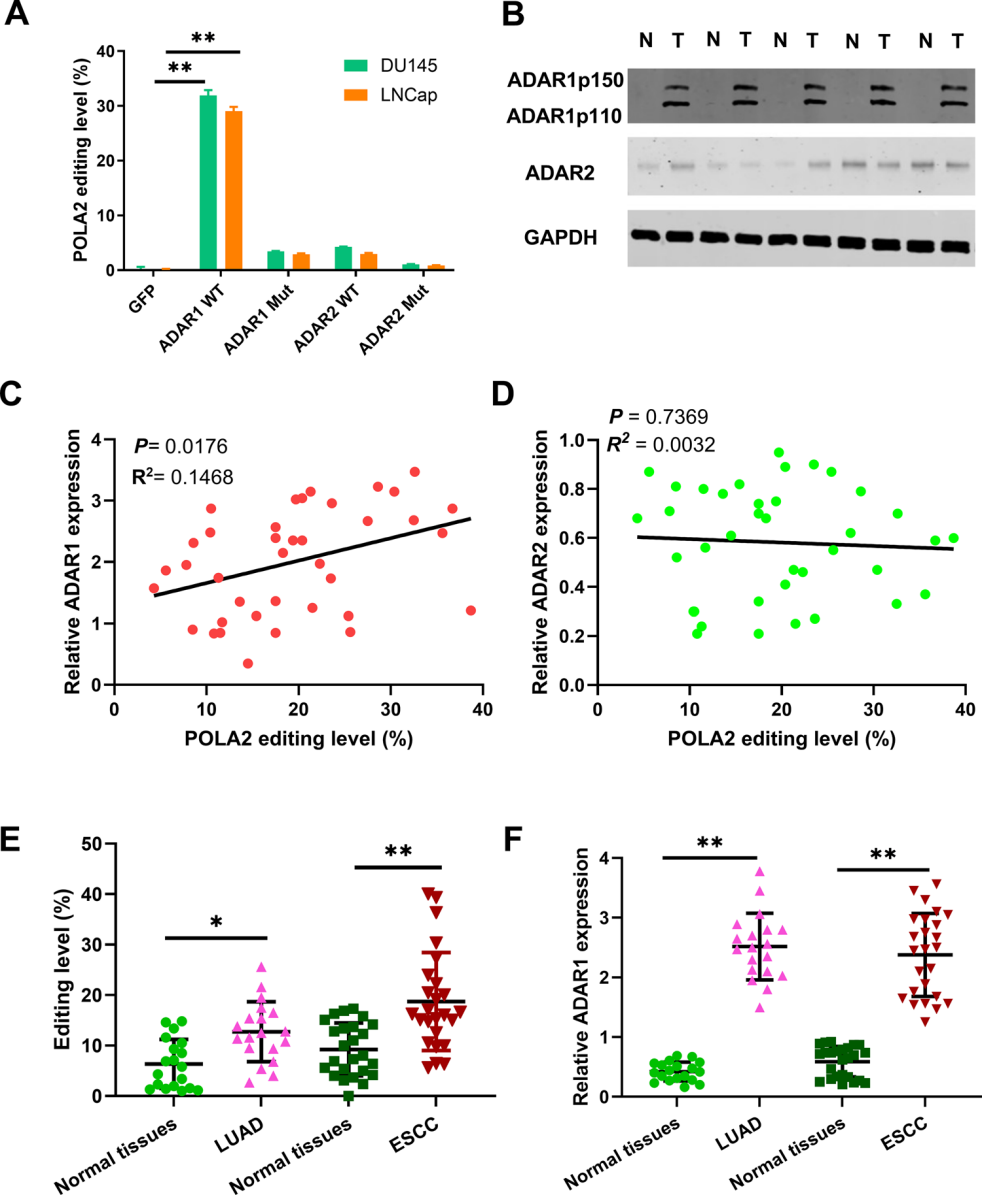
A-to-I RNA editing of POLA2 is mediated by ADAR1 enzyme in cancers. **A** The editing level changes of POLA2 in DU145 and LNCap cells after transfection of wild-type ADAR enzymes (ADAR WT) or inactive ADAR enzymes (ADAR mut). **B** ADAR1 and ADAR2 expression in normal and PCa tissues. ADAR1p110 and ADAR1p150 are the two ADAR1 transcript variants, encoding 110-kDa (p110) and 150-kDa (p150) isoforms. **C**, **D** Correlation between POLA2 editing level and the relative expression of ADAR1 or ADAR2 in PCa tissues. **E** POLA2 editing level in normal, LUAD and ESCC tissues. **F** ADAR1 expression in normal, LUAD and ESCC tissues. *P < 0.05, **P < 0.01

To explore how A-to-I RNA editing of POLA2 occurs in PCa, ADAR1 or ADAR2 enzyme was transfected into DU145 and LNCap PCa cell lines as it has been proved that ADAR1 and ADAR2 is the main catalytic enzyme for A-to-I RNA editing. ADAR perturbation experiment showed that the editing level of POLA2 was obviously enhanced in PCa cells carrying ADAR1-WT, but was not affected by ADAR1-Mut, ADAR2-Mut and ADAR2-WT (Fig. 2A). At the same time, high expression of ADAR1 (ADAR1-p110 and ADAR1-p150) was detected in PCa tissues in comparison to normal tissues (Fig. 2B). However, ADAR2 expression was not significantly different between PCa and normal tissues (Fig. 2B). Additionally, we found that POLA2 editing level was positively correlated with ADAR1 expression, but did not have association with ADAR2 expression in PCa patients (Fig. 2C, D). To further confirm the effect of ADAR1 on POLA2 editing in other cancers, POLA2 editing and ADAR1 expression in esophageal squamous cell carcinoma (ESCC) and lung adenocarcinoma (LUAD) were determined. Similarly, high POLA2 editing and ADAR1 expression were also identified in LUAD and ESCC tissues compared to normal tissues (Fig. 2E, F). These findings reveal that A-to-I RNA editing of POLA2 is mainly mediated by ADAR1 enzyme in human cancers.

### POLA2 overediting aggravates malignant behaviors of PCa

**Fig. 3:**
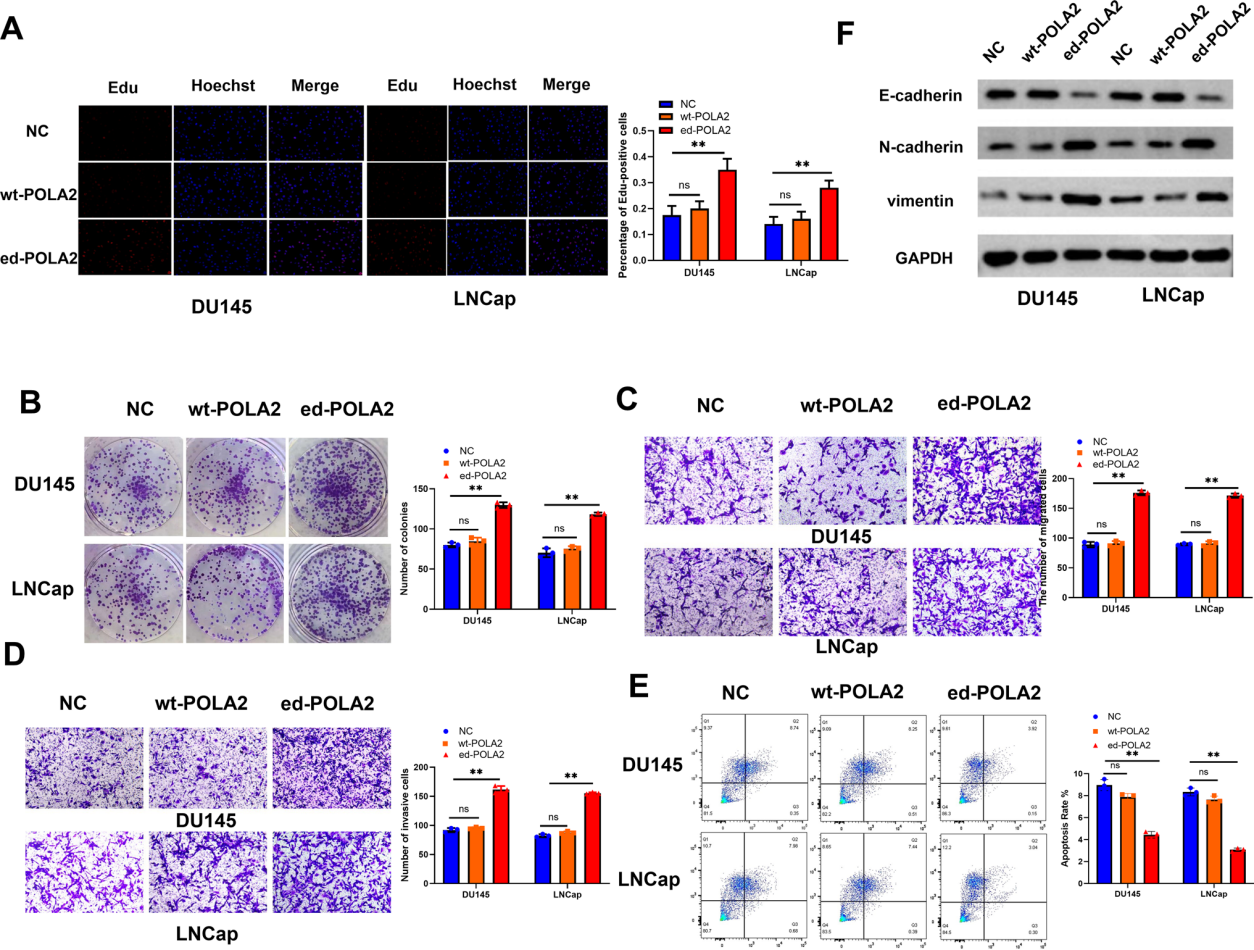
POLA2 overediting aggravates malignant behaviors of PCa. **A**, **B** The proliferation of DU145 and LNCap cells containing wild-type or edited POLA2 vector. **C**, **D** The migration and invasion of DU145 and LNCap cells containing wild-type or edited POLA2 vector. **E** The apoptosis of DU145 and LNCap cells containing wild-type or edited POLA2 vector. **F** The protein expression of E-cadherin, N-cadherin, vimentin in DU145 and LNCap cells containing wild-type or edited POLA2 vector. **P < 0.01

Next, wild-type (wt-POLA2) or edited POLA2 (ed-POLA2) vector was transfected into DU145 and LNCap cells to investigate the role of wild-type or edited POLA2 in PCa. Compared to NC group, the proliferation of DU145 and LNCap cells was not affected by wt-POLA2, but was dramatically accelerated by ed-POLA2 (Fig. 3A, B). The similar effects of wt-POLA2 or ed-POLA2 on PCa cell migration and invasion were also identified (Fig. 3C, D). Moreover, we found that PCa cell apoptosis rate was reduced in ed-POLA2 group, but had little change in wt-POLA2 group compared to NC group (Fig. 3E). Besides, the effect of wt-POLA2 and ed-POLA2 on EMT markers E-cadherin, N-cadherin, vimentin was investigated in PCa cells. As showed in Fig. 3F, E-cadherin protein expression was observed to be declined by ed-POLA2, but was not affected by wt-POLA2 in both DU145 and LNCap cells. The protein expressions of N-cadherin and vimentin were enhanced by ed-POLA2, but not affected by wt-POLA2 in PCa cells (Fig. 3F). Taken together, A-to-I RNA editing endowers POLA2 with carcinogenicity in PCa development, and POLA2 overediting aggravates cell viability and metastasis of PCa.

### POLA2 overediting fortifies glycolysis of PCa cells.

**Fig. 4:**
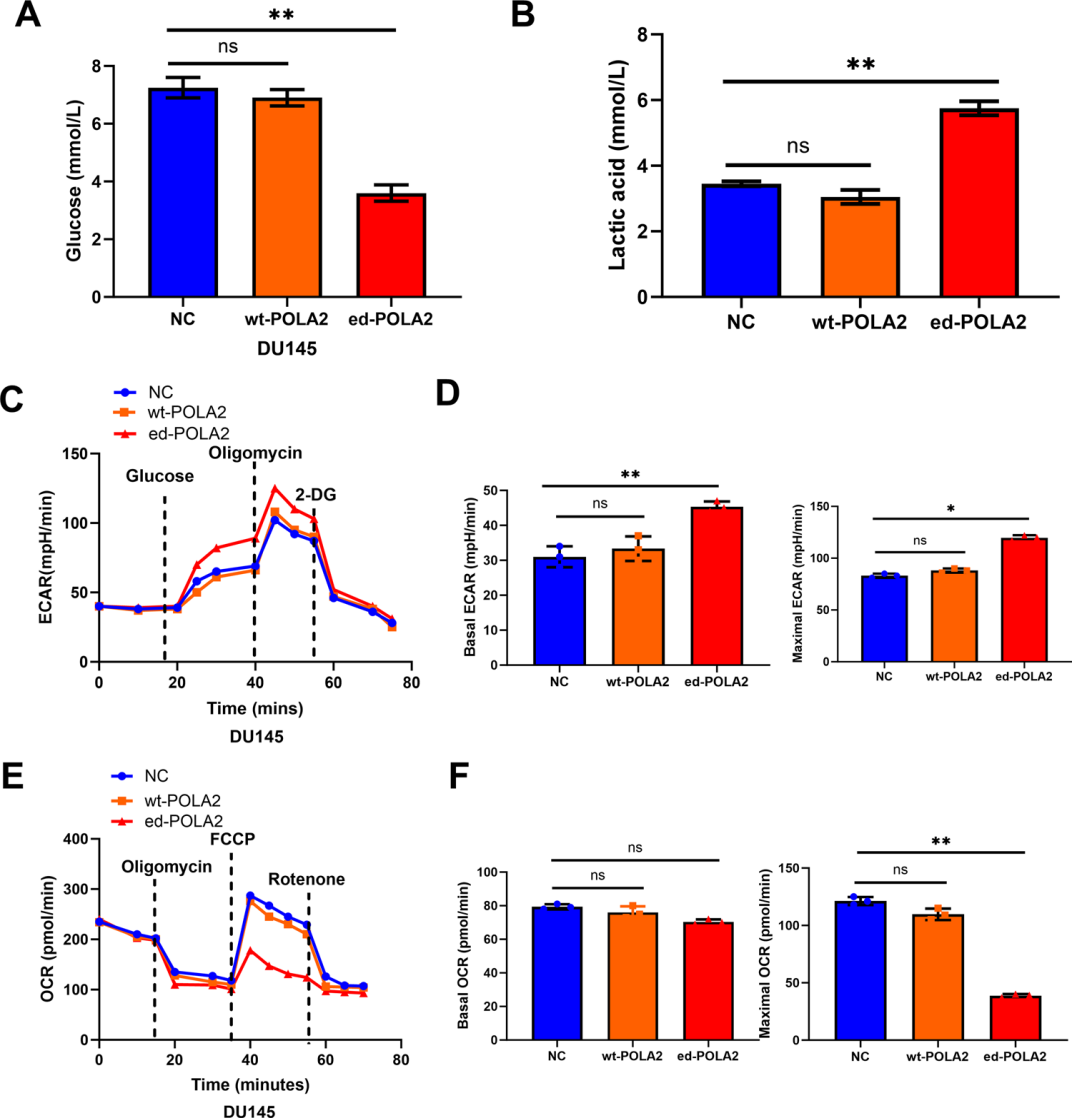
POLA2 overediting fortifies glycolysis of PCa cells. **A**, **B** Lactic acid and glucose level in DU145 cells containing wild-type or edited POLA2 vector. **C** The representative curves of ECAR in DU145 cells containing wild-type or edited POLA2 vector. **D** Quantification of basal and maximal ECAR. **E** The representative curves of OCR in DU145 cells containing wild-type or edited POLA2 vector. **F** Quantification of basal and maximal OCR. *P < 0.05, **P < 0.01

To explain how POLA2 overediting triggers tumorigenesis of PCa, the effect of POLA2 overediting on glycolysis of PCa cells was assessed by OCR and ECAR. First, we found that DU145 cells with ed-POLA2 exhibited decreased glucose level and increased lactic acid level compared to NC (Fig. 4A, B). The ECAR analysis detected by Seahorse XF96 Analyzer showed that both the basal and maximal ECAR were increased in ed-POLA2 group compared to NC and wt-POLA2 groups (Fig. 4C, D). Moreover, POLA2 overediting dramatically declined the maximal OCR of PCa cells compared to NC and wt-POLA2 groups (Fig. 4E, F). Moreover, there were no significant differences in lactic acid level, glucose level, ECAR and OCR between wt-POLA2 group and NC group (Fig. 4A–F). All above results demonstrate that POLA2 overediting exerts carcinogenic effect in PCa by fortifying glycolysis.

### Edited POLA2 upregulates BTBD7 expression in PCa by binding to miR-596

**Fig. 5:**
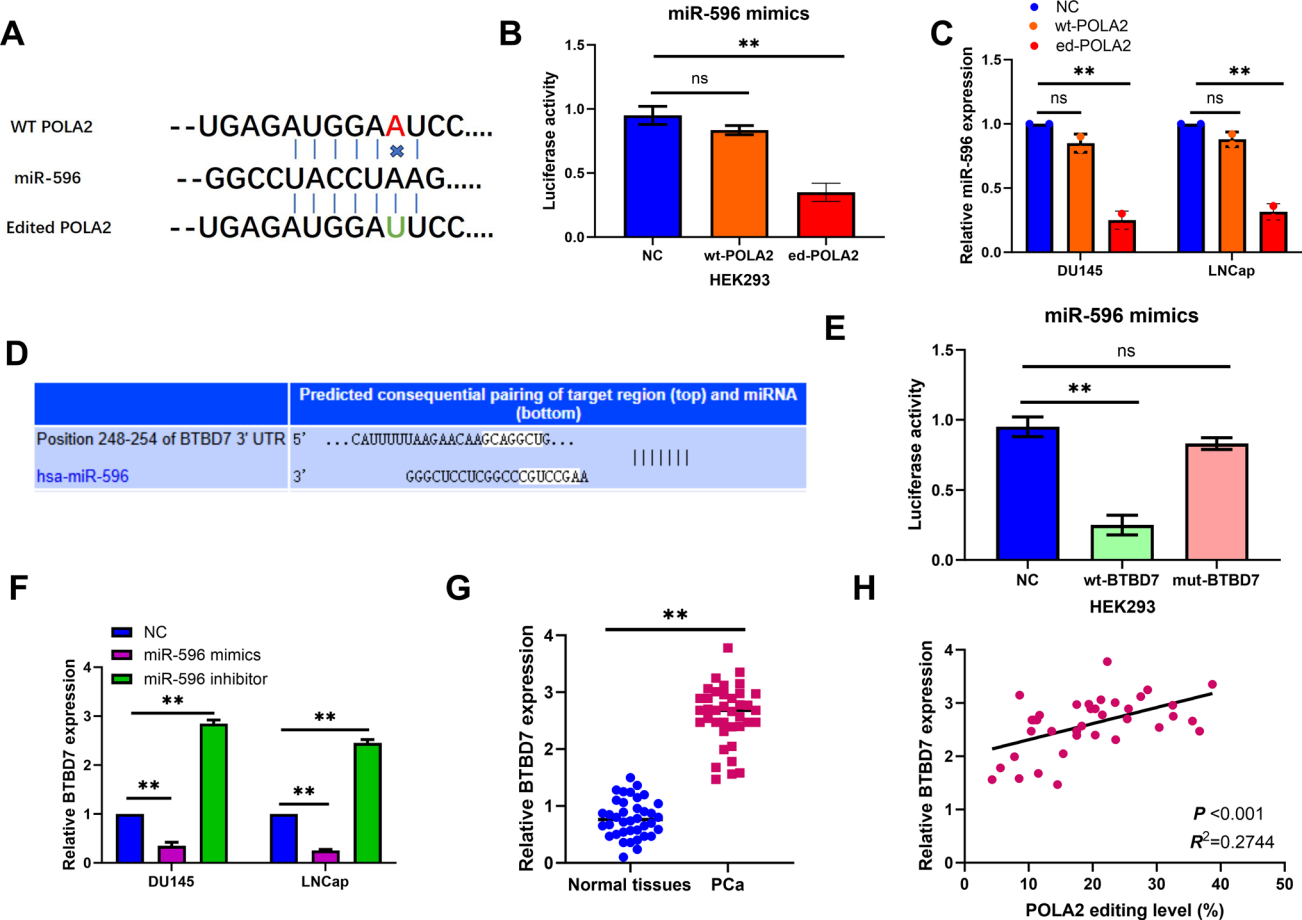
Edited POLA2 upregulates BTBD7 expression in PCa by binding to miR-596. **A** The binding site between edited POLA2 and miR-596. **B** Dual-luciferase reporter assay was designed to confirm the relationship between edited POLA2 and miR-596. **C** The expression of miR-596 in DU145 and LNCap cells containing wild-type or edited POLA2 vector. **D** The binding site between BTBD7 and miR-596. **E** Dual-luciferase reporter assay was designed to confirm the relationship between BTBD7 and miR-596. **F** The expression of BTBD7 in DU145 and LNCap cells containing miR-596 mimics or inhibitor. **G** The expression of BTBD7 in normal and PCa tissues. **H** Correlation between the relative expression of BTBD7 and POLA2 editing level in PCa tissues. **P < 0.01

Mechanically, CAeditome database (https://ccsm.uth.edu/CAeditome/index.html) predicted that edited POLA2 obtains a new binding site with miR-596 (Fig. 5A). Luciferase reporter assay displayed that luciferase activities of ed-POLA2 were reduced by miR-596 mimics in HEK293 cells, but miR-596 mimics did not affect wt-POLA2 luciferase activity (Fig. 5B). At the same time, miR-596 expression was found to be reduced by ed-POLA2, but was not affected by wt-POLA2 in PCa cells (Fig. 5C). Furthermore, BTBD7 was predicted to be a potential target of miR-596 by TargetScanHuman 7.2 (https://www.targetscan.org/vert_72/, Fig. 5D). And this prediction was confirmed by luciferase reporter assay (Fig. 5E). Besides that, miR-596 mimics reduced BTBD7 expression, while miR-596 inhibitor enhanced BTBD7 expression in LNCap and DU145 cells (Fig. 5F). Compared to normal tissues, upregulation of BTBD7 was found in PCa tissues (Fig. 5G). In PCa tissues, a positive correlation was detected between BTBD7 expression and POLA2 editing level (Fig. 5H). These findings reveal that edited POLA2 upregulates BTBD7 expression in PCa by binding to miR-596.

### POLA2 overediting stimulates PCa tumor growth in vivo

**Fig. 6:**
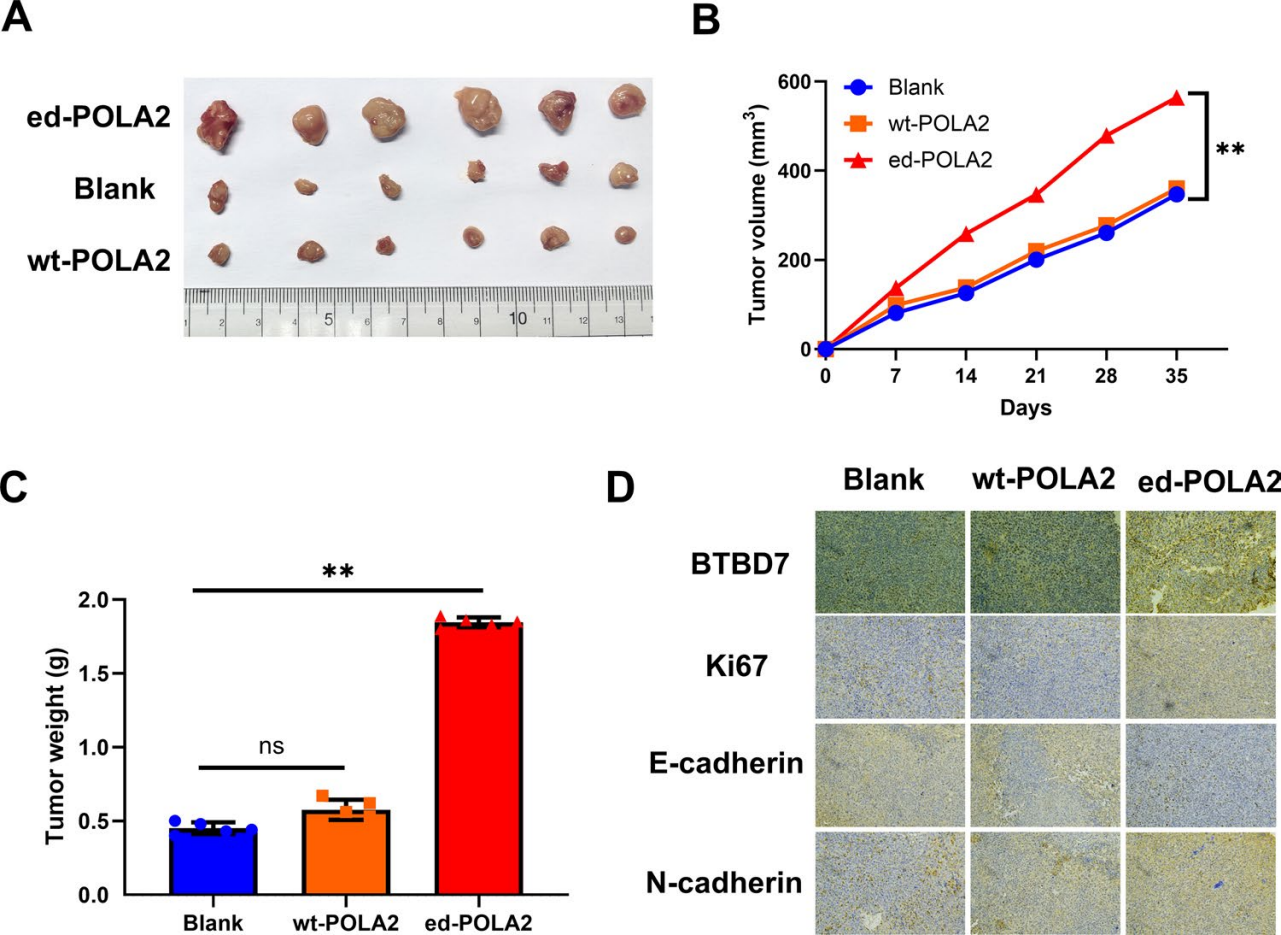
POLA2 overediting stimulates PCa tumor growth in vivo. **A** Representative tumor images. **B** Tumor volume in Blank, wt-POLA2 and ed-POLA2 groups. **C** Tumor weight in Blank, wt-POLA2 and ed-POLA2 groups. **D** BTBD7, E-cadherin, N-cadherin and Ki67 expression in mice tissues of Blank, wt-POLA2 and ed-POLA2 groups detected by Immunohistochemistry assay. **P < 0.01

Then, in vivo mice models were established to further confirm the role of POLA2 editing in PCa. As shown in Fig. 6A, tumor size is larger in ed-POLA2 group compared to wt-POLA2 and Blank groups. And there was almost no difference in tumor size between wt-POLA2 group and Blank group (Fig. 6A). Additionally, tumors grew obviously faster in ed-POLA2 group than those in wt-POLA2 and Blank groups (Fig. 6B). Correspondingly, heavier tumor weight was measured in ed-Myo19 group in comparison with Blank and wt-POLA2 groups (Fig. 6C). Moreover, Immunohistochemistry assay suggested that the positive rate of BTBD7 and Ki67 was increased in mice tissues of ed-POLA2 group compared with Blank group, but there was little difference in positive rate of BTBD7 between wt-POLA2 group and Blank group (Fig. 6D). Collectively, POLA2 overediting stimulates PCa tumor growth in vivo by upregulating BTBD7.

### A-to-I RNA edited POLA2 impairs CD8 + T cell cytotoxicity in PCa

**Fig. 7:**
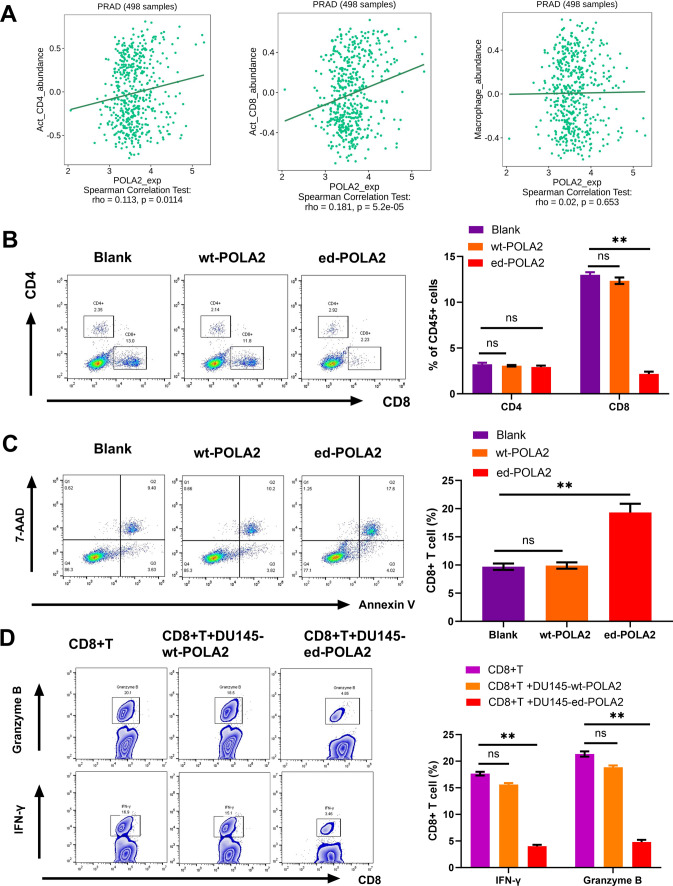
A-to-I RNA edited POLA2 impairs CD8+ T cell cytotoxicity in PCa. **A** The correlation between POLA2 expression and the abundance of tumor-infiltrating CD8+ T cells and CD4+ T cells. **B** FACS analysis of the percent of tumor-infiltrating T cells in mice tissues of Blank, wt-POLA2 and ed-POLA2 groups. **C** CD8+ T cell apoptosis in mice tissues of Blank, wt-POLA2 and ed-POLA2 groups. **D** FACS analysis of Granzyme B and IFN-γ of CD8+ T cells treated with DU145 cells containing wt-POLA2 or ed-POLA2 vector. **P < 0.01

Additionally, the bioinformatics analysis of TISIDB database (http://cis.hku.hk/TISIDB/) showed that POLA2 expression was significantly correlated with the abundance of tumor-infiltrating CD8+ T cells and CD4+ T cells, but had no correlation with macrophage abundance in PCa (Fig. 7A). Based on the above results, we inferred that POLA2 editing may also influence immune microenvironment in PCa. Just as we predicted, Flow cytometry analysis showed the decreased frequency of CD8+ T cells in mice tumor containing ed-POLA2 compared to mice tumors in Blank and wt-POLA2 groups (Fig. 7B). Meanwhile, POLA2 overediting was also found to enhance CD8+ T cell apoptosis in comparison with Blank and wt-POLA2 groups (Fig. 7C). Additionally, CD3/CD28-activated T cells were co-cultured with DU145 cells containing ed-POLA2 vector. We found that CD8+ T cells co-cultured with DU145 cells containing ed-POLA2 produced less Granzyme B and IFN-γ compared to CD8+ T cells without treatment and CD8+ T + DU145-wt-POLA2 group (Fig. 7D). Summarily, A-to-I RNA edited POLA2 can hinder immune response in PCa by impairing CD8+ T cell cytotoxicity.

## Discussion

A-to-I RNA editing is an in-depth study of RNA editing, which is widespread in mammals. Numerous A-to-I RNA editing events have been identified in the human cancer transcriptome recently, and many of them are clinically relevant [24]. Since post-transcriptional regulation is complex, it is unclear how A-to-I RNA editing affects cancer development. In this study, A-to-I RNA overediting of POLA2 was identified in PCa patients, which was related to tumor stage, tumor recurrence and bad prognosis. The role of A-to-I RNA editing POLA2 has not been investigated in any human cancers and is firstly confirmed in PCa. In additionally, we found the high expression of ADAR1 in PCa tissues. ADAR1 is a member of ADAR family, whose gene sequence is highly conserved in vertebrates [25]. And it has been demonstrated that the A-to-I RNA editing enzymes ADAR1 highly expressed in PCa cells DU145 and PC3 cells [26]. Our research also showed that POLA2 editing levels was positively related to ADAR1 expression in PCa tissues. The above findings reveal that A-to-I RNA editing of POLA2 is mediated by ADAR1 enzyme in PCa. Besides that, high expression of ADAR1 and POLA2 overediting was also detected in LUAD and ESCC tissues, indicating that ADAR1 is the key enzyme that causes POLA2 editing in tumors.

Functionally, POLA2 overediting aggravated cell viability and metastasis of PCa. The carcinogenesis of POLA2 has been reported in hepatocellular carcinoma [27] and lung cancer [28]. However, the function of wild-type and edited POLA2 has not been investigated in PCa. Our study found that wild-type POLA2 had no significant effect on PCa development. A-to-I RNA editing endowered POLA2 with carcinogenicity in PCa development. It has been reported that A-to-I RNA editing can cause changes in gene function, gene structure and biological signal transduction pathway [24], which supports our results. Going further, we confirmed that edited POLA2 obtains a new binding site with miR-596, and wt-POLA2 had little effect on miR-596 expression. Dai et al. has proposed that overexpression of miR-596 suppressed PCa cell growth and migration [29]. Combining the fact that edited POLA2 downregulated miR-596 in PCa, we considered that edited POLA2 expedited PCa progression by suppressing miR-596 expression. Additionally, this study also confirmed that miR-596 directly targets BTBD7. And upregulation of BTBD7 was detected in PCa tissues, which was positively associated with POLA2 editing level. These results indicate that edited POLA2 upregulates BTBD7 expression in PCa by binding to miR-596. Chen et al. has reported that BTBD7 served as a tumor promoter in PCa [23]. These findings reflect that edited POLA2 promotes PCa progression by upregulating BTBD7 through competitively binding to miR-596.

In terms of metabolic mechanism, edited POLA2 overediting was found to fortify glycolysis. As we all known, a common feature of tumor cells is that glucose intake and lactic acid accumulation gradually increase even under the condition of normal oxygen content. Tumor cells use glycolysis as the main source of energy metabolism, and obtain higher glycolytic ability [30]. Here, we found that DU145 cells with ed-POLA2 exhibited decreased glucose level and increased lactic acid level, indicating that ed-POLA2 enhanced the ability of PCa glycolysis. Moreover, many genes have been found to control PCa tumor growth by regulating glycolysis, such as HOXA11-AS [31] and GLDC [32]. However, it is firstly reported that the relationship between ed-POLA2 stimulates tumorigenesis of PCa by encouraging glycolysis. Additionally, bioinformatics analysis showed that POLA2 expression was closely related to the abundance of tumor-infiltrating CD8+ T cells and CD4+ T cells, but had no correlation with macrophage abundance in PCa. On the contrary, Teng et al. reported that POLA2 upregulation is correlated with macrophage infiltration in hepatocellular carcinoma [18]. These findings prove that the effect of POLA2 on tumor immune microenvironment is changeable. Therefore, we suspect that A-to-I RNA editing may also induce the change of POLA2 effect on PCa immune microenvironment. Consistent with our prediction, edited POLA2 only influenced the tumor-infiltration of CD8+ T cells and impaired CD8+ T cell cytotoxicity in PCa. Taken together, A-to-I RNA edited POLA2 can hinder immune response in PCa by impairing CD8+ T cell cytotoxicity, thus promoting PCa development. Nevertheless, there are still many shortcomings in this study, such as limited sample size. Moreover, this study only preliminarily explained the regulatory mechanism of edited POLA2 in PCa, and its more complex regulatory network is still unclear and needs to be explored.

## Conclusion

In conclusion, A-to-I RNA overediting of POLA2 in PCa patients was related to unfavorable clinical outcomes and prognosis. A-to-I RNA editing of POLA2 mediated by ADAR1 enzyme attained carcinogenesis in PCa. More importantly, edited POLA2 overediting fortified glycolysis and impaired CD8+ T cell cytotoxicity in PCa. Mechanically, edited POLA2 triggered tumorigenesis of PCa by upregulating BTBD7 through competitively binding to miR-596. Our research provides new insights into the pathogenesis of PCa and sheds light on the mechanisms involved in A-to-I RNA editing in tumors.

## Data Availability

The datasets used and/or analyzed during the current study are available from the corresponding author on reasonable request.
